# Effect of Seed Sludge Type on Aerobic Granulation, Pollutant Removal and Microbial Community in a Sequencing Batch Reactor Treating Real Textile Wastewater

**DOI:** 10.3390/ijerph191710940

**Published:** 2022-09-01

**Authors:** Jinte Zou, Jiaqi Yang, Hangtian He, Xiaofei Wang, Rongwu Mei, Lei Cai, Jun Li

**Affiliations:** 1 Key Laboratory of Microbial Technology for Industrial Pollution Control of Zhejiang Province, Zhejiang University of Technology, Hangzhou 310014, China; 2 College of Environment, Zhejiang University of Technology, Hangzhou 310014, China; 3 Center for Microbial Ecology and Technology (CMET), Ghent University, Coupure Links 653, 9000 Ghent, Belgium; 4 Eco-Environmental Science Design & Research Institute of Zhejiang Province, Hangzhou 310007, China

**Keywords:** aerobic granular sludge, real textile wastewater, seed sludge, removal of COD and nitrogen, microbial community

## Abstract

The aerobic granulation, pollutant removal, and microbial community in real textile wastewater (TWW) treatment were compared using conventional activated sludge (CAS) and preformed aerobic granular sludge (AGS) in synthetic wastewater as seed in two reactors, reactor-1 (R1) and reactor-2 (R2), respectively. The results showed that complete granulation was achieved in R1 (sludge volume index at 5 min (SVI_5_) and 30 min (SVI_30_): 19.4 mL/g; granule size: 210 μm) within 65 days, while it only required 28 days in R2 (SVI_5_ and SVI_30_: 27.3 mL/g; granule size: 496 μm). The removal of COD, NH_4_^+^-N and TN in R1 (49.8%, 98.8%, and 41.6%) and R2 (53.6%, 96.9%, and 40.8%) were comparable in 100% real TWW treatment, but stable performance was achieved much faster in R2. The real TWW had an inhibitory effect on heterotrophic bacteria activity, but it had no inhibition on ammonia-oxidizing bacteria activity. AGS with a larger particle size had a higher microbial tolerance to real TWW. Furthermore, filamentous *Thiothrix* in the AGS in R2 disappeared when treating real TWW, leading to the improvement of sludge settleability. Thus, seeding preformed AGS is suggested as a rapid start-up method for a robust AGS system in treating real TWW.

## 1. Introduction

The treatment of the effluents of textile industries is extremely challenging and important due to the high concentration of recalcitrant and toxic compounds such as dyes, surfactants, heavy metal ions, detergents, and solvents [1,2]. Previously, physical and/or chemical processes such as the Fenton reaction, ozonation adsorption, and persulfate activation have been used to treat textile wastewater (TWW). However, novel biological processes with eco-friendly, effective, and economical features are in urgent need [3,4]. Aerobic granular sludge (AGS) has been extensively studied in recent years for municipal and industrial wastewater treatment [5,6]. Compared to conventional activated sludge (CAS), AGS features a number of advantages such as more compact microbial structure, higher biomass content, better settling performance, and the ability to withstand a higher organic loading rate (OLR) and toxic pollutants [7,8,9].

Up to now, there have only been a few studies on the application of AGS for the treatment of TWW, and most of them have been carried out with synthetic TWW. Muda et al. first reported the development of AGS in dye-containing synthetic wastewater using mixed sludge (sewage, textile mill sludge, and anaerobic granules) as seed [10]. Their subsequent study noted that both the hydraulic retention time (HRT) and the length of the anaerobic phase affected the AGS properties and removal of color and chemical oxygen demand (COD) [11]. This is mainly due to the removal of azo dyes by N=N bond cleavage in the anaerobic stage and generation of aromatic amines, which are mineralized in the aerobic stage [12]. The layered structure of AGS from outside to inside leads to the difference in oxygen distribution, and AGS can achieve azo-dye decolorization in the in the anaerobic inner core during the aerobic phase of a sequencing batch reactor (SBR) cycle [1]. Ma et al. cultured stable AGS in synthetic wastewater containing methylene blue, and the removal efficiency of methylene blue reached 56% [12]. Franca et al. studied the influence of Acid Red 14, an azo dye, on an AGS system, and found that AGS can adapt to the environment of azo dye and achieved a removal efficiency of over 90% in a 1.5 h anaerobic reaction phase [13]. Sarvajith et al. achieved 89–100% stable removal of azo dye after 80 days of operation under microaerophilic conditions [14].

Compared to synthetic TWW, the composition of real TWW has higher complexity and variability. Limited studies have been conducted on using AGS for real TWW treatment, and this application is not yet well explored. Lotito et al. investigated the effectiveness of a sequencing batch biofilter granular reactor for the pre-treatment of real TWW and the treatment of mixed municipal-textile wastewater [15,16]. However, the AGS properties and granulation process were not deeply investigated in their studies. Ibrahim et al. and Kee et al. studied AGS formation in real TWW [3,17]. However, its application seems to be difficult because the real TWW had been sterilized before feeding and four dye-degrading bacteria were used for the AGS formation. Manavi et al. found that AGS formed in synthetic media disintegrated during long-term exposure to real TWW [1]. Bashiri et al. reported that AGS formed in slaughterhouse wastewater was able to treat real TWW, but AGS collapsing and activity reduction occurred in 100% real TWW [18]. In these two studies, microbial community analysis was not involved. In fact, there are limited studies investigating the changes of nitrifying bacteria activity and microbial community during sludge granulation in real TWW treatment. Additionally, forming AGS in TWW is time-consuming, which may restrict its application. Previous studies have reported that the formation of AGS in synthetic TWW required 21–87 days while it needed from 6 weeks to 300 days in real TWW [3,12,17,18,19]. Hence, it is meaningful to study the rapid start-up of AGS systems in real TWW.

In this study, aerobic granulation, pollutant removal, and microbial community in real TWW treatment process were compared, with the application of two types of seed sludge, CAS from wastewater treatment plant (WWTP) and preformed AGS in synthetic wastewater. Biomass concentration, sludge settleability and morphology, particle size, extracellular polymeric substances (EPS) content, reactor performance, and microbial activity and community were monitored during the experimental period. The results of the present study will further help to understand the use of AGS technology for real TWW treatment.

## 2. Materials and Methods

### 2.1. Experimental Set-Up and Operation

Two identical plexiglass SBRs (R1 and R2) with a working volume of 3.0 L and an effective height-to-diameter ratio of 2.9 were used. The volumetric exchange ratio was maintained at 50% with an HRT of 12 h. The SBRs were operated with a 6-h cycle consisting of static feeding (10 min), anaerobic stir (110 min), aeration (180 min), settling (R1: 30 min on days 0–20, 20 min on days 21–42, 5 min on days 43–72; R2: 10 min on days 0–8, 5 min on days 9–35), decanting (5 min), and idling (25–50 min corresponding to the settling). Bottom aeration was supplied with an airflow rate of 3.0 L/min. The temperature was controlled at 25 ± 2 °C with a circulating water jacket.

R1 was inoculated with CAS taken from an aerobic tank of a municipal WWTP in Hangzhou, China. Since the granulation time in R1 was long, R2 was inoculated with preformed AGS after 33 days of running of R1. The preformed AGS was taken from the laboratory SBR treating synthetic wastewater (705 mg/L of sodium acetate, 152.8 mg/L of NH_4_Cl, and 26.3 mg/L of KH_2_PO_4_, corresponding COD, ammonia nitrogen (NH_4_^+^-N) and phosphorus were 550, 40, and 6 mg/L). The characteristics of seed CAS and seed AGS are shown in Table 1. During the experimental period, biomass wastage was not controlled in the two reactors but occurred through sludge washout during the decanting phase.

### 2.2. Wastewater Composition

The real TWW was collected every 2–3 weeks from the hydrolysis acidification tank of a textile company in Shaoxing, China. The raw TWW was settled for about 24 h before use. A high variability in the composition of real TWW was observed in this experiment. The concentrations of COD, NH_4_^+^-N, and total nitrogen (TN) fluctuated in the range of 400–1300, 80–170, and 100–200 mg/L, respectively. To allow biomass acclimatization, the percentage of real TWW in the influent (dilution with tap water) gradually increased from 50% to 100% (R1: 50% on days 0–33, 70% on days 34–56, 100% on days 57–72; R2: 50% on days 0–7, 70% on days 8–19, 100% on days 20–35) [18]. Additionally, 128 mg/L sodium acetate (100 mgCOD/L) was added to the influent because of the low BOD_5_/COD ratio (0.15–0.33) in the raw TWW. Furthermore, 21.9 mg/L KH_2_PO_4_ (5 mgP/L) was added to the influent because of the phosphorus deficiency in the raw TWW. The resulting COD/N/P ratio of influent was around 100:20:1. The pH value of the influent was around 7.0–8.0.

### 2.3. Analytical Methods

COD, NH_4_^+^-N, NO_3_^−^-N, NO_2_^−^-N, TN, mixed-liquor suspended solids (MLSS), mixed-liquor volatile suspended solids (MLVSS), and sludge volume index (SVI_5_ and SVI_30_) were measured according to the standard methods [20]. The pH value was measured by a multi-parameter analyzer (Multi 3420, WTW, Weilheim, Germany). Sludge particle size was monitored using a laser particle analyzer (Mastersizer 3000, Malvern, UK). D10, D50, and D90 indicated that 10%, 50%, and 90% of the total particle volume had a smaller particle size than the values of D10, D50, and D90, respectively. Sludge EPS was extracted using a heating method [9]. The content of polysaccharides (PS) in EPS was determined by the anthrone-sulfuric acid method and the content of proteins (PN) in EPS was determined by a bicinchoninic acid protein assay kit [9]. The specific oxygen uptake rate (SOUR) of bacteria (including ammonia-oxidizing bacteria (AOB), nitrite-oxidizing bacteria (NOB), and heterotrophic bacteria (HB)) in R1 and R2 were measured following the method described by Zou et al. [21]. The morphology of sludge was observed using an optical microscope (CX31, Olympus, Tokyo, Japan).

### 2.4. Microbial Community Analysis

Sludge samples were collected including seed CAS, seed AGS, sludge on day 40 and 72 in R1 (R1-40d and R1-72d), and sludge on day 22 in R2 (R2-22d). High-throughput Illumina Miseq sequencing analysis for these sludge samples was conducted at Zhejiang Tianke Hi-Tech, Inc. (Hangzhou, China). A PowerSoil^®^ DNA Isolation Kit (MoBio, San Diego, CA, USA) was used for DNA extraction. The DNA quality was checked on a 1% (*w*/*w*) agarose gel and the DNA samples were quantified using a NanoDrop2000 Fluorospectrometer (Thermo Scientific, Wilmington, DE, USA). Then, V3 and V4 regions of the bacterial 16S rDNA sequence were amplified by polymerase chain reaction (PCR) with the primers of 341F (5′-CCTACGGGNGGCWGCAG-3′) and 805R (5′-GACTACHVGGGTATCTAATCC-3′) [22]. PCR amplification was performed in triplicate in 30 μL mixture containing 15 μL of 2 × Phusion Master Mix, 1.5 μL of each prime (2 μM), 10 μL of template DNA (1 ng/μL), and 2 μL of H_2_O. The thermal program was as follows: hot start 98 °C for 1 min, followed by 30 cycles of denaturation (98 °C for 10 s), annealing (50 °C for 30 s), extension (72 °C for 30 s), and a final extension at 72 °C for 5 min. Amplicons were extracted from 2% (*w*/*w*) agarose gels and purified using a GeneJET Gel Extraction Kit (Thermo Scientific, Wilmington, DE, USA), and quantified using a NanoDrop2000 Fluorospectrometer (Thermo Scientific, Wilmington, DE, USA). The purified amplicons from each reaction mixture were pooled in equimolar and paired-end sequenced (2 × 250) on an Illumina Miseq platform (Miseq PE300, Illumina, San Diego, CA, USA) according to the standard protocols. After sequencing, data were collected as follows: (i) reads were truncated at any site receiving an average quality score < 20 over a 10 bp sliding window; (ii) exact barcode matching, two nucleotide mismatches in primer matching, and reads containing ambiguous characters were removed; (iii) only sequences that overlapped by longer than 10 bp were assembled according to their overlap sequence. Reads that could not be assembled were discarded. Operational taxonomic units (OTUs) were clustered with a 97% identity threshold using Uparse. The phylogenetic affiliation of each 16S rDNA sequence was analyzed by an RDP Classifier against the Silva 16S rDNA database using confidence threshold of 70% [22]. The Good’s coverage and alpha diversity (Shannon and Simpson indexes) were calculated following the method described by Good [23] and Magurran [24].

## 3. Results and Discussion

### 3.1. Evolution of Sludge Characterization

#### 3.1.1. Formation of AGS in R1

**Figure 1:**
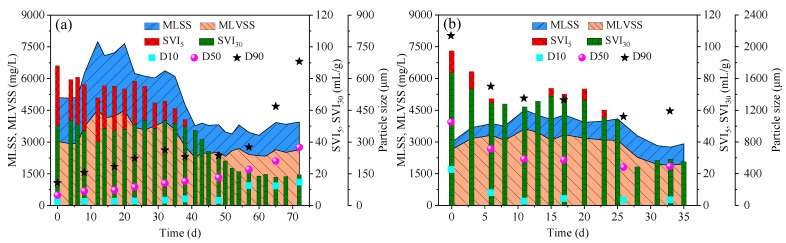
Variations in MLSS, MLVSS, SVI_5_, SVI_30_, and sludge particle size in R1 (**a**) and R2 (**b**) throughout the operational period.

**Figure 2:**
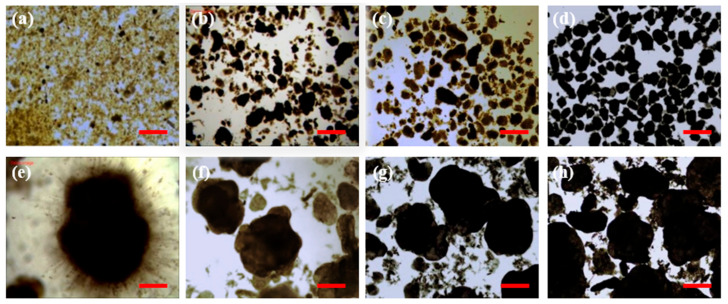
Microscope images of sludge in R1 (seed conventional activated sludge (**a**), on day 38 (**b**), day 61 (**c**), and day 68 (**d**)) and R2 (seed aerobic granular sludge (**e**), on day 9 (**f**), day 19 (**g**), and day 29 (**h**)) (scale bar (red) = 500 μm).

As shown in Figure 1a, in the first 20 days, the MLSS in R1 increased from 5102 mg/L to 7654 mg/L, and correspondingly, the MLVSS increased from 3024 mg/L to 4508 mg/L. The reason for the increase was probably due to the long settling time (30 min) and relatively high removed rate of COD (0.87 ± 0.11 g/(L·d)) in this period [25]. When the settling time gradually decreased to 5 min and the COD removed rate declined to 0.51 ± 0.17 g/(L·d), the MLSS and MLVSS accordingly decreased to 3686 ± 228 mg/L and 2497 ± 126 mg/L, respectively. The SVI_5_ gradually declined to 54.1 mL/g and the ratio of SVI_30_/SVI_5_ reached 0.92 on day 38 for the first time. After that, the sludge settleability was further improved as indicated by the further decrease in SVI_5_ and SVI_30_ (19.4 ± 1.3 mL/g). The ratio of SVI_30_/SVI_5_ was always maintained at 1.00 from day 41 to day 72. The sludge particle size increased slowly during the AGS cultivation using real TWW. After 65 days of operation, D50 gradually increased from 49 μm to 210 μm and D10 and D90 also increased by over four times. The formation of AGS in R1 was also supported by the evolution of sludge morphology (Figure 2). The seed CAS started with small particle size and loose structure when inoculated in R1 (Figure 2a). The number of sludge aggregates grew noticeably and some AGS with a large particle size and dense structure were observed on day 38 (Figure 2b). After 61–68 days of cultivation, AGS with clear outline and dense structure became the dominant form in R1 (Figure 2c,d). When the SVI_30_/SVI_5_ ratio and mean sludge particle size were greater than 0.9 and 200 μm, respectively, and a clear outline of the sludge was observed, granulation was considered to be completed [26,27]. Therefore, it was assumed that complete granulation was achieved after about 65 days of cultivation in the real TWW treatment by seeding CAS. The time consumption for forming AGS from CAS was consistent with the results reported previously (granulation time of 42–112 days using real TWW) [1,3,17].

**Figure 3:**
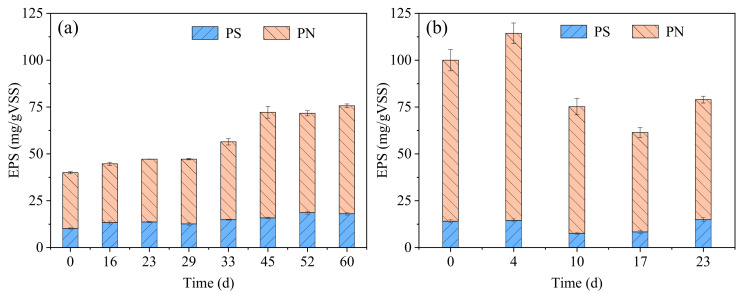
Changes in sludge EPS content (PN and PS) in R1 (**a**) and R2 (**b**).

EPS is secreted by bacterial consortia during cell metabolism and PN and PS are demonstrated as the major constituents in EPS [28]. In R1, the PS content in the EPS gradually increased from 10.2 mg/(gVSS) (day 0) to 18.0 mg/(gVSS) (day 60), and correspondingly, the PN content in the EPS increased from 29.8 mg/(gVSS) to 57.7 mg/(gVSS) (Figure 3a). It is known that EPS plays a major role in the aggregation of microorganisms, granule formation, and structure stability [8]. The increasing content of EPS helped to promote the conversion of CAS into AGS in the real TWW treatment.

#### 3.1.2. Formation of AGS in R2

As shown in Figure 1b, in the first 11 days, the MLSS in R2 increased from 2988 mg/L to 4514 mg/L, and correspondingly, the MLVSS increased from 2636 mg/L to 3616 mg/L. After that, the MLSS and MLVSS gradually declined to 2831 ± 80 mg/L and 1932 ± 24 mg/L, respectively. The SVI_5_ and SVI_30_ for the seed AGS were 97.4 mL/g and 83.7 mL/g respectively, and the ratio of SVI_30_/SVI_5_ was 0.86, illustrating that the seed AGS had poor settleability. This was attributed to the overgrowth of filamentous bacteria in the seed AGS (Figure 2e). However, the SVI_5_ and SVI_30_ both decreased to 64.0 mL/g and the ratio of SVI_30_/SVI_5_ increased to 1.00 on day 8 (Figure 1b). This could be owing to the rapid disappearance of filamentous overgrowth in the AGS treating real TWW (Figure 2f–h). From day 15 to day 23, the SVI_5_ and SVI_30_ increased slightly, but the ratio of SVI_30_/SVI_5_ was always greater than 0.9 (Figure 1b). After that, the SVI_5_ and SVI_30_ both dropped to 27.3 ± 2.1 mL/g. These results indicate that excellent sludge settleability could be rapidly achieved in real TWW treatment by seeding AGS within 28 days. Additionally, the granule size exhibited a decreasing trend in the first 11 days, illustrating the occurrence of AGS breakage. Visual and microscopic observations of granule fragments also supported this conclusion (Figure 2f). The decrease in granule size was possibly related to the adaptation to the real TWW, which was also observed in other studies treating real TWW and simulated pharmaceutical wastewater [15,29]. Interestingly, the granule size remained relatively stable after 12 days of operation even when the percentage of real TWW reached 100% (Figure 1b). This was not consistent with the previous study, where AGS formed in slaughterhouse wastewater collapsed in 100% real dyeing wastewater [18]. Overall, compact AGS with excellent sludge settleability (SVI_5_ and SVI_30_: 27.3 ± 2.1 mL/g) and a stable granule size (496 ± 18 μm) was successfully achieved within 28 days, 57% less time consuming compared with using CAS as seed (65 days). Seeding preformed AGS in synthetic wastewater can be used as a strategy for the rapid start-up of AGS systems treating real TWW.

The seed AGS preformed in synthetic wastewater had a higher EPS content (100.0 mg/(gVSS)) than the seed CAS (35.9 mg/(gVSS)) and AGS formed in R1 (75.7 mg/(gVSS)) (Figure 3). After 4 days of cultivation, the PN content in the EPS increased from 85.9 mg/(gVSS) to 99.9 mg/(gVSS) and the PS content in the EPS remained stable (Figure 3b). A previous study reported that bacteria secrete more EPS to protect themselves from a harsh environment [30]. However, with further operation, the contents of PN and PS in the EPS both decreased to 53.1 mg/(gVSS) and 8.3 mg/(gVSS), respectively. A similar phenomenon was also observed in other studies treating real TWW for a long time using AGS and this was explained as the consumption of EPS as energy and carbon sources by microorganisms [1,31]. It is interesting that the stable EPS contents in R1 and R2 were at the same level. This may imply that the EPS content in the AGS system was related to the wastewater characterization.

### 3.2. Removal of COD, NH_4_^+^-N and TN

**Figure 4:**
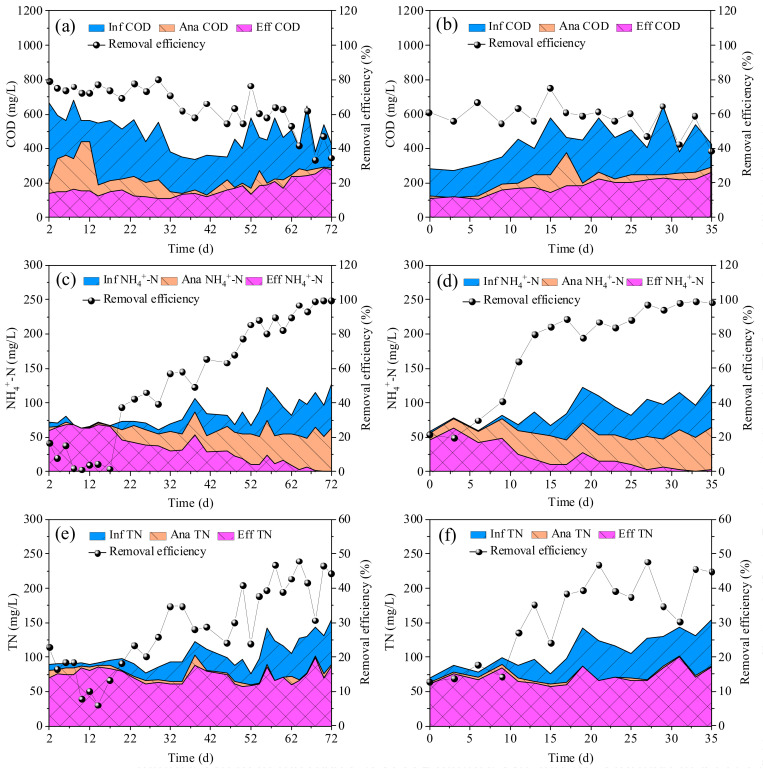
Variations of COD, NH_4_^+^-N, and TN in R1 (**a**,**c**,**e**) and R2 (**b**,**d**,**f**) throughout the operational period.

**Figure 5:**
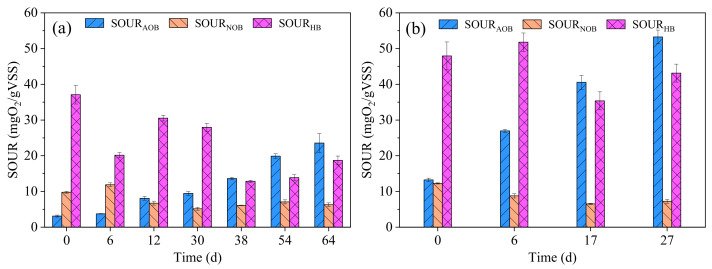
Variations of SOUR_AOB_, SOUR_NOB_ and SOUR_HB_ in R1 (**a**) and R2 (**b**) throughout the operational period.

It should be noted that the COD concentration in the influent (including the COD in real TWW and added acetate) did not increase with the increasing percentage of real TWW (Figure 4). This was mainly due to the change in the production process and reduction in the output of the textile company, leading to a decrease in COD concentration in the raw TWW at the later stages of the experiment. In R1, the average COD removal was 74.6% in the first 33 days (50% of real TWW) (Figure 4a). It decreased to 61.4% on days 34–56 (70% of real TWW). On days 57–72, it further decreased to 49.8% (100% of real TWW). The average COD removal decreased with the increased proportion of real TWW. This indicates that some refractory organics in the real TWW could not be degraded under the present conditions. In R2, the COD removal remained at approximately 61% in the first 19 days (Figure 4b). Increasing the percentage of real TWW from 50% to 70% did not affect the COD removal. However, the average COD removal decreased to 53.6% with a further increase to 100% real TWW (days 20–35). R2 did not show a significant advantage over R1 with regard to the COD removal. The average COD removal in R1 (49.8%) and R2 (53.6%) was lower than those reported by Lotito et al. (62.1% at HRT 31.2–52.8 h) and Manavi et al. (68% at HRT 34.3 h) treating real TWW using AGS [1,15]. The short HRT (12 h), especially the short anaerobic HRT (3.7 h), in this study was probably the main reason for the low COD removal, as refractory organics degradation needed a longer HRT [11]. In addition, in R1, the SOUR_HB_ on day 6 (20.2 mgO_2_/(gVSS)) was lower than the seed CAS (37.1 mgO_2_/(gVSS)) (Figure 5a), indicating that the real TWW had an inhibition effect on HB activity. The inhibition of HB activity became weaker as the sludge adapted to the environment, as indicated by the increase in SOUR_HB_ on day 12 (30.5 mgO_2_/(gVSS)). With the increasing percentage of real TWW to 70%, the SOUR_HB_ declined again on day 38 (12.9 mgO_2_/(gVSS)). However, after complete granulation, the SOUR_HB_ on day 64 (18.7 mgO_2_/(gVSS)) increased slightly even when the percentage of real TWW increased to 100%. This indicates that AGS has high tolerance to toxic inhibitors present in real TWW, corresponding to results reported previously [5]. Moreover, the SOUR_HB_ of AGS in R2 (43.1 mgO_2_/(gVSS) on day 27) was higher than that in R1 (18.7 mgO_2_/(gVSS) on day 64). This is mainly attributed to the fact that the granule size in R2 was much larger than that in R1 (Figure 1 and Figure 2). In fact, AGS with a larger particle size had a higher substrate mass transfer resistance, resulting in a higher tolerance to toxic substances present in real TWW [5,32].

The NH_4_^+^-N removal in R1 (1.2–16.6%) was very low in the initial period, and then increased gradually with sludge adaptation (Figure 4c). After 68 days of operation, the NH_4_^+^-N removal reached 98.8%, corresponding to the SOUR_AOB_ increase from 3.1 mgO_2_/(gVSS) (day 0) to 23.6 mgO_2_/(gVSS) (day 64) (Figure 5a). AOB activity in R1 was thus not inhibited by the application of real TWW. Similar variation trends in NH_4_^+^-N removal and SOUR_AOB_ were also observed in R2 (Figure 4d and Figure 5b). However, the NH_4_^+^-N removal was higher than 90% after 64 days in R1, while it only required 27 days in R2. In addition. the SOUR_AOB_ in R2 (53.3 mgO_2_/(gVSS) on day 27) was higher than that in R1 (23.6 mgO_2_/(gVSS) on day 64) (Figure 5). These results indicate that R2 showed an advantage over R1 with regard to the NH_4_^+^-N removal. Contradictory to the increasing SOUR_AOB_, the SOUR_NOB_ in R1 and R2 decreased slightly at first, and then stabilized at 6.3 ± 0.7 mgO_2_/(gVSS) and 7.5 ± 1.2 mgO_2_/(gVSS), respectively. Consequently, partial nitrification was observed in both reactors. The main form of nitrogen in effluent of R1 and R2 were nitrite, and the average nitrite accumulation ratio in R1 and R2 reached 79.0% and 81.8%, respectively (Figure S1). The occurrence of partial nitrification was owing to the high free ammonia (FA) concentration in the initial aerobic phase in R1 (6.1–11.7 mg/L) and R2 (5.9–9.9 mg/L) (data not shown). Generally, the inhibition dose of FA for NOB is 0.1–1.0 mg/L while it is 10–150 mg/L for AOB [33]. Additionally, the TN removal in R1 decreased to 7.9% in the initial period due to the worsening of the NH_4_^+^-N removal (Figure 4c,e). After that, it gradually increased. The average TN removal under the steady-state conditions in R1 was 41.6% (days 54–72). Similarly, it was 40.8% (days 21–35) in R2. The low TN removal was mainly due to the high TN concentration and insufficient biodegradable carbon sources in the influent. As discussed above, refractory organics were difficult to degrade under the present conditions and the amount of removed COD decreased over time (Figure 4a,b). Furthermore, the TN concentrations at the end of anaerobic phase were almost the same as those in the effluent, indicating that simultaneous nitrification–denitrification (SND) did not occur in R1 and R2. This might also have contributed to the low TN removal. It should be pointed out that according to the equation of oxygen penetration depth, anoxic zones were present in the aeration phase in R2 due to the large granule size (481–1050 μm) [34,35]. Therefore, no occurrence of SND was attributed to the insufficient biodegradable organics in the real TWW.

### 3.3. Microbial Community Characterization

It was revealed that 37891 (seed CAS), 28908 (R1-40d), 25413 (R1-72d), 41023 (seed AGS), and 32173 (R2-22d) effective reads were obtained from the five sludge samples after interception, filtering, and removal of chimeric sequences, with a yield of 90.7%, 87.9%, 74.9%, 90.9%, and 84.2%, respectively. The alpha diversity indexes for different sludge samples are shown in Table 2. The values of Good’s coverage in all the sludge samples were greater than 99.5%, indicating that the microbial community could be well represented by the collected gene sequences [9]. A decrease in microbial diversity was observed in R1 as indicated by the decrease in the Shannon and Simpson indexes from 6.85 and 0.932 in seed CAS to 4.83 and 0.859 in R1-72d. This might be due to the washout of microorganisms during the granulation period and the inhibition of bacterial growth caused by the real TWW [36,37]. However, the microbial diversity did not decrease in R2 as indicated by the similar values of the Shannon and Simpson indexes in the seed AGS (5.45 and 0.920) and R2-22d (5.52 and 0.950). This was probably due to the high tolerance of the AGS to toxic substances, contributing to the maintenance of microbial diversity in the real TWW [5,32].

**Figure 6:**
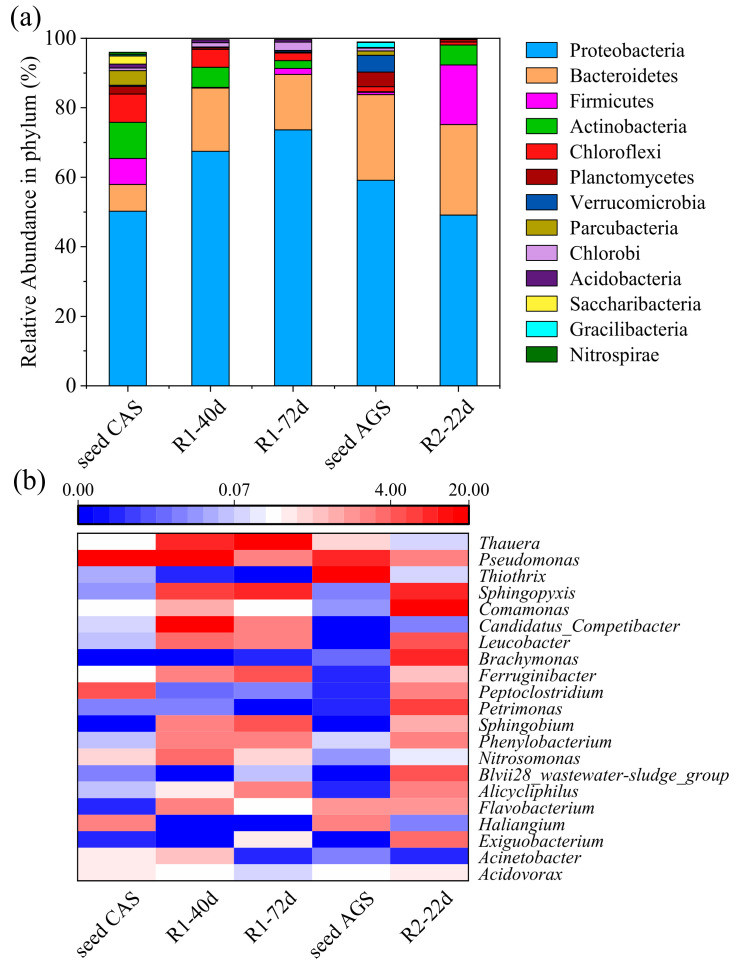
Microbial community characterization for seed CAS, sludge of R1-40d and R1-72, seed AGS, and sludge of R2-22d ((**a**) relative abundance at the phylum level; (**b**) richness heat map of the microbial community at the genus level).

As shown in Figure 6a, the dominant phyla in the seed CAS were Proteobacteria (50.2%), Bacteroidetes (7.7%), Firmicutes (7.5%), Actinobacteria (10.3%), and Chloroflexi (8.1%). After 72 days of running of R1, the relative abundances of Proteobacteria and Bacteroidetes increased to 73.6% and 15.9%, respectively, while the relative abundances of the rest of the phyla decreased to 1.7%, 2.3%, and 2.1%, respectively. Generally, Proteobacteria is predominant in wastewater treatment processes and includes many functional bacteria that are capable of degrading different kinds of pollutants and secreting EPS to promote sludge granulation [8,22,38]. Similarly, Proteobacteria and Bacteroidetes were also the dominant phyla in the seed AGS (59.1% and 24.7%) and R2-22d (49.1% and 26.1%). However, the relative abundance of Firmicutes in R2 significantly increased from 0.7% to 17.1% after 22 days of operation, as did the relative abundance of Actinobacteria (from 0.1% to 5.8%). As reported previously, Firmicutes can produce endospores to enhance tolerance against environmental stress [39]. Actinobacteria possess a firmed-structure and higher-adaptability, which could maintain sludge structure [40].

At the genus level, *Pseudomonas* was predominant in the seed CAS (24.7%), while the relative abundance of *Pseudomonas* decreased to 1.2% in R1-72d (Figure 6b). A similar phenomenon (from 8.4% to 1.3%) was also observed in R2. *Pseudomonas* was reported to have the function of potential denitrification [8]. In addition, other genera possessing potential denitrification functions such as *Flavobacterium*, *Haliangium*, *Acidovorax*, and *Acinetobacter* had low relative abundances (<1%) in R1-72d and R2-22d [41,42]. These might be related to the low TN removal in R1 and R2. The relative abundance of *Thauera* increased dramatically from 0.2% in the seed CAS to 39.1% in R1-72d, and the relative abundance of *Comamonas* increased from 0.0% in the seed AGS to 15.2% in R2-22d. As reported previously, *Thauera* and *Comamonas* can secret excessive EPS that is crucial for sludge granulation, structure stability, and resistance in a harsh environment [8,42,43]. *Thiothrix* is a common filamentous bacterium [35], which was predominant in the seed AGS (24.3%) but was hardly found in R2-22d (0.1%). This was consistent with the results of sludge morphology and sludge settleability in R2 (Figure 1b and Figure 2e,f). As described by Kouki et al., *Exiguobacterium* has an NH_4_^+^-N oxidation ability under environmental stress conditions [44]. Therefore, the relative abundances of AOB (*Nitrosomonas* and *Exiguobacterium*) in R1-72d (0.8%) and R2-22d (3.7%) were higher than those in the seed CAS (0.4%) and seed AGS (0.1%). Moreover, the relative abundance of NOB (*Nitrospira*) was hardly observed in R1-72d and R2-22d. These were consistent with the results, in that partial nitrification occurred in R1 and R2 (Figure 5 and Figure S1).

## 4. Conclusions

Complete granulation in real TWW was achieved much faster in R2 (28 days; SVI_5_ and SVI_30_: 27.3 mL/g; D50: 496 μm) by seeding preformed AGS than in R1 (65 days; SVI_5_ and SVI_30_: 19.4 mL/g; D50: 210 μm) by seeding CAS. The removal performance of COD, NH_4_^+^-N, and TN removal in 100% real TWW treatment were comparable in R1 (49.8%, 98.8%, and 41.6%) and R2 (53.6%, 96.9%, and 40.8%), but stable performance was achieved much faster in R2. The real TWW had an inhibitory effect on HB activity, but it had no inhibition on AOB activity. AGS with larger particle size had a higher microbial tolerance to the real TWW. Moreover, filamentous *Thiothrix* in AGS in R2 disappeared when treating real TWW, resulting in the improvement of sludge settleability. Thus, seeding preformed AGS is suggested as a rapid start-up method for a robust AGS system treating real TWW.

## Figures and Tables

**Table 1:** Characteristics of seed conventional activated sludge (CAS) and seed aerobic granular sludge (AGS).

Parameters	Seed CAS	Seed AGS
SVI_30_ (mL/g)	50.3	83.0
SVI_5_ (mL/g)	88.1	96.5
MLSS (mg/L)	5102	2988
MLVSS (mg/L)	3024	2636
Mean particle size (μm)	48.6	1050
Polysaccharides (PS) in EPS (mg/(gVSS))	10.2 ± 0.4	14.1 ± 0.9
Proteins (PN) in EPS (mg/(gVSS))	29.8 ± 0.5	85.9 ± 5.6

**Table 2:** The alpha diversity indexes for different sludge samples.

Samples	OUTs	Shannon	Simpson	Coverage (%)
seed CAS	828	6.852	0.932	99.66
R1-40d	397	6.218	0.968	99.74
R1-72d	387	4.828	0.859	99.56
seed AGS	397	5.447	0.920	99.76
R2-22d	343	5.523	0.950	99.69

## Data Availability

The data presented in this study are available in the article and Supplementary Materials.

## Supplementary Materials

The following supporting information can be downloaded at: https://www.mdpi.com/article/10.3390/ijerph191710940/s1, Figure S1: Variations of NO_3_^−^-N and NO_2_^−^-N in the influent, at the end of anaerobic phase, and in the effluent and variation in the nitrite accumulation ratio in R1 (a) and R2 (b) throughout the operational period.
