# Supplementary material for: Effect of Seed Sludge Type on Aerobic Granulation, Pollutant Removal and Microbial Community in a Sequencing Batch Reactor Treating Real Textile Wastewater
Source: Int J Environ Res Public Health. 2022 Sep 1;19(17):10940. doi: 10.3390/ijerph191710940 (PMC9518340; doi:10.3390/ijerph191710940)
Supplement: Supplementary file 1 [file ijerph-19-10940-s001.zip › Figure S1.pdf]

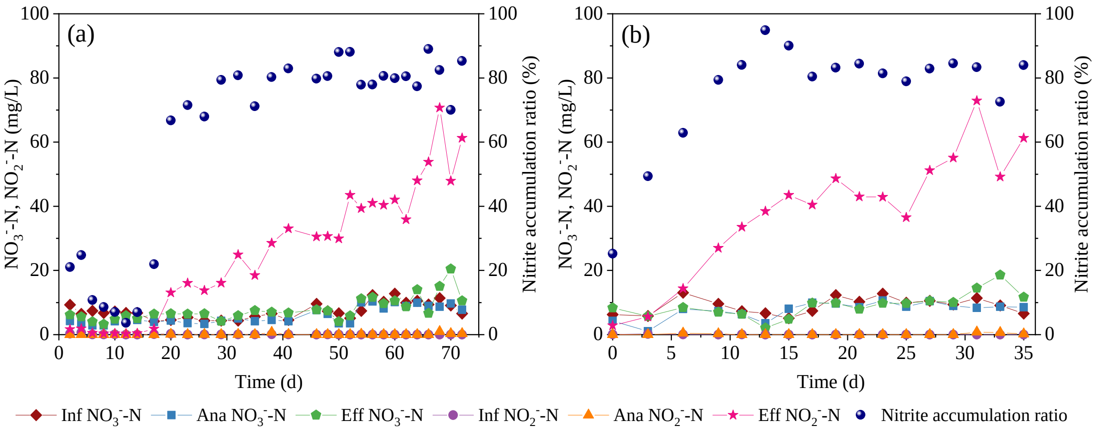

Figure S1 Variations of NO_3^--N and NO_2^--N in the influent, at the end of anaerobic phase, and in the effluent and variation in the nitrite accumulation ratio in R1 **(a)** and R2 **(b)** throughout the operational period.
